# Use of Health Services Among People Living Alone in Finland

**DOI:** 10.1177/11786329211043955

**Published:** 2021-09-01

**Authors:** Pia CM Solin, Tytti P Pasanen, Katariina AJ Mankinen, Tuija P Martelin, Nina M Tamminen

**Affiliations:** Equality Unit, Finnish Institute for Health and Welfare, Helsinki, Finland

**Keywords:** Living alone, use of health services, mental health

## Abstract

Although health issues are more common in people living alone than in those living with someone, research on the service use of people living alone has focused on older age groups. Based on large Finnish cross-sectional health survey (FinHealth 2017, n = 4686), we examined the difference in the use and assessment of health services between those living alone and those living with someone, and whether some sub-groups within those living alone use or perceive the use of health care services differently to those living with someone. The adjusted proportions, based on logistic regression models controlling for demographic variables and perceived health and mental health, showed that those living alone had seen a doctor in the past year less often (65.5%) than those not living alone (71.9%). People living alone had also less often had a health examination in the past 5 years (72.4%) than those not living alone (79.2%), and this proportion was particularly low within people living alone with high levels of depressive symptoms (59.0%) compared to lower levels (75.0%). Conclusively, among people living alone, those who suffer from depressive symptoms might be a potential group that does not receive the same levels of preventive care than others.

## Background

Living alone has become more common in today’s societies. In 2018, one third (33.9%) of households in the European Union were single-person households.^1^ In Finland, there are 1.2 million people living alone, which is almost 45% of all house-hold dwelling units are 1 person households.^2^ Despite the high number of people living alone, research on mental health and health care service use of people living alone has been largely focused on the elderly and their well-being. These studies have found that retired individuals living alone use less preventive care than those who live with a partner.^3,4^ We do not know whether these issues apply to people living alone in all age groups or if they are specific to older age groups. However, we do know that poverty seems to be common among those living alone. Low resources have a negative effect on following a healthy diet, taking part in activities and getting services, which otherwise would be accessible.^5^ Furthermore, on average, satisfaction with their state of health was only average and lower compared with those who live with a partner.^6,7^ Mental health problems were especially identified as one of the most essential factors that have a negative effect on the state of health of those living alone.^7^ Furthermore, it was found that one third of those living alone were forced to compromise their use of health services and medicine due to poor economic situation.^6^ A Finnish study found that those living alone were approximately twice as likely to have anxiety or depressive disorder compared to the married.^6^

The Finnish healthcare system is based on public healthcare services to which everyone in the country is entitled. The Constitution of Finland^8^ guarantees that the public authorities shall provide adequate social, health, and medical services for everyone. There are also numerous private healthcare services in Finland. Moreover, employers must offer all their employees occupational health care, aiming at preventing work-related illnesses and accidents, promoting safety, and maintaining the health of workers. In addition to preventive health care, an employer may also arrange for medical services for their employees.^9^ To our knowledge, despite weaker health and mental health status of those living alone, health care services do not give special focus on people living alone, for example, in preventative measures or lifestyle counseling.

The first aim of the present paper is to examine the difference in the use and assessment of health services between those living alone and those living with someone, controlling for the known differences (ie, perceived general and mental health) in the need of these services. The second aim is to assess whether some sub-groups within those living alone, based on demographic or mental health characteristics, use or perceive the use of health care services differently to those living with someone. With this approach, we can assess whether there are certain sub-groups within people living alone that are at risk of not receiving adequate support.

## Methods

### Data

The questionnaire data from the FinHealth 2017 Study was used. The study is a nationally representative health examination survey carried out in 2017 in Finland.^10^ Altogether 10 305 persons (of whom 10 247 were eligible) aged 18 or over, selected based on a stratified, probability proportional to size, sampling design, were invited to participate in a health examination and to fill in several questionnaires.^10^ For this study, information from 2 main self-report questionnaires were used: the first which was sent to the whole sample and returned at the health examination (or by mail), and the second which was collected only from those who participated in the health examination.^11^ The second questionnaire included questions about the use and assessment of health services and their accessibility. In total, 5337 persons (52.1% of the eligible sample, and 89.2% of those who participated in the health examination) returned this questionnaire.^12^ Due to some missing responses, the sample size in regression analyses varied between 2514 and 4686 (see Table 1).

**Table 1.** Proportion or mean (SD) of the study variables in respondents living alone and with someone (sample restricted to those who responded in the second questionnaire, and weighed according to non-respondence and clustered sampling design).

Variable	Category	Not living alone (n = 4021)	Living alone (n = 1290)
Gender (n = 5311)	Male	46.5	37.1
	Female	53.5	62.9
Age (n = 5311)	18-29	7.1	10.0
	30-64	64.4	45.7
	65-99	28.5	44.3
Perceived health (n = 5295)	Average/poor	31.6	44.7
	Good/rather good	68.4	55.3
Participation in societies/clubs (n = 5268)	None	42.5	42.8
	Sometimes	22.1	22.9
	Actively	35.4	34.4
Education (n = 5222)	Low	31.5	32.5
	Middle	33.8	31.8
	High	34.7	35.6
MHI5 (n = 5097)	Low	94.5	91.1
	High	5.5	8.9
BDI6 (n = 5072)	Low	90.6	85.0
	High	9.4	15.0
WEMWBS (n = 5068)	Low	12.3	20.0
	Average	73.4	67.5
	High	14.3	12.6
Doctor’s appointment in the past 12 mo (n = 5217)	No	27.3	26.5
	Yes	72.7	73.5
Nurse’s appointment in the past 12 mo (n = 5212)	No	51.9	49.3
	Yes	48.1	50.7
Health examination in the past 5 y (n = 5186)	No	20.6	27.5
	Yes	79.4	72.5
Used health services because of mental health problems in the past 12 mo (n = 5213)	No	93.6	91.1
	Yes	6.4	8.9
Positive experiences of primary care (n = 3984)	No	43.9	47.9
	Yes	56.1	52.1
Positive experiences of the access to primary care (n = 2739)	No	42.2	51.6
	Yes	57.8	48.4
Social relationship index (4-19) (n = 5100)		9.9 (0.05)	9.5 (0.09)
Equivalized household income (thousands) (n = 5125)		35.0 (0.3)	27.3 (0.5)

### Measures

#### Health service use and evaluation of health services—dependent variables

Health service use was assessed with the following questions: (1) the frequency of doctor’s appointments in the past 12 months, (2) the frequency of nurse’s appointments in the past 12 months, (3) the frequency of used health services because of mental health problems in the past 12 months, (4) the frequency of health examinations in the past 5 years, (5) positive experiences of primary care, and (6) positive experiences of the access to primary care. Measures 1 to 4 were dichotomized (has used/not used the service), and measures 5 and 6 were formed by dichotomizing the means of 4 and 6 items (each rated on a scale from 1 = always to 4 = never), respectively, evaluating different aspects of these experiences. A positive experience was determined as the average of 1.5 or less, indicating having always had a positive experience on most aspects.

#### Living alone—predictor

Living alone was defined as living in a household of only 1 member. In the data, among those who had responded to at least some of the use of health services items, 1285 (24%) persons reported to live alone.

#### Mental health and mental well-being—predictors/moderators

Mental health and mental well-being were measured by means of the Warwick-Edinburgh Mental Well-Being Scale (WEMWBS), the Mental Health Inventory (MHI-5), and Beck Depression Inventory (BDI-6).

There are several overlapping concepts in the area of mental healthiness and thus it is important to clarify the concepts of *mental wellbeing* and *mental health. Mental wellbeing* describes positive states of being, thinking, behaving, and feeling, thus it is the counterpart of PMH. *Mental health* is a concept which describes a range of states from good mental health to severe mental health problems in a linear fashion.^13^ Furthermore, PMH bases on the idea that mental health and mental ill-health situate in 2 separate continuums. Therefore, mental health is more than absence of a diagnosed mental health disorder.^14,15^

A validated Warwick-Edinburgh Mental Well-Being Scale (WEMWBS) measures positive mental well-being. It measures positive functioning (energy, clear thinking, self-acceptance, personal development, mastery, and autonomy), satisfying interpersonal relationships, and positive feeling (feelings of optimism, cheerfulness, relaxation) with 14 items. These Likert-style scales produce a single score and record person’s statements about their thoughts and feelings over the past 2 weeks with positively phrased questions. The items are rated with “None of the time”; “Rarely”; “Some of the time”; “Often” and “All of the time.”^14^ The total score (sum of all items) was grouped into low (14-43), average (44-61), and high (62-70) WEMWBS based on sample mean and standard deviation, following established conventions.^16^

The Mental Health Inventory-5 (MHI-5) is a valid and reliable instrument for assessing both psychological well-being and distress.^17,18^ MHI-5 measures the presence of psychological well-being and the absence of psychological distress with 5 questions, each with 6 possible responses; “All of the time”; “Most of the time”; “A good bit of time”; “Some of the time”; “A little of the time” and “None of the time.” The sum of these items was scaled to 1 to 100 points. A cut-off at 52 points was used for grouping the respondents into 2 classes, in line with prior population reports identifying this as a threshold for potential clinical psychological distress.^13,19,20^

The 6-item version of the Beck Depression Inventory (BDI-6), was developed from the original 21-item BDI by Aalto et al.^21^ BDI-6 assesses depressed mood, pessimism, dissatisfaction, guilt, self-dislike, and indecisiveness. BDI was, likewise, grouped into low and high depressive symptoms with a cut-off at 4 points, according to prior reports establishing this as indicative of acute depressive symptoms.^13,21^

#### Socio-demographic and behavioral factors—covariates

The selected covariates were socio-demographic and behavioral factors known to affect mental health, health behavior, and the use of health services (eg, Koponen et al^22^). These included age, sex (binary), self-rated health (on a scale of 1-5 with smaller values indicating better health; specified as a factor), participation in activities provided by organizations or societies (never, sometimes, and often; specified as a factor), educational level (a factor with low, average, and high levels, based on years of educational attainment, adjusted for gender and age group), household income per consumption unit, low/high MHI-5 score, and an index of social relationships. The social relationship index ranged from 4 to 19, and it was created by summing up values of 4 variables measuring how often one is in contact with friends and relatives in person, by phone, or over the Internet (each on a scale from 1 = never to 5 = daily) and the amount of close friends (on a scale from 1 = none to 4 = more than 2). Descriptive statistics of the covariates as well as the 3 mental health measures are presented in Table 1 among people not living alone and those living alone.

### Analysis strategy

The adjusted proportions for the use of services were estimated by predictive margins method^23^ using the survey package in the R statistical software version 3.6.0.^24^ The adjustment models were binary logistic regression models with each service use variable as a response, adjusted for the following covariates: age, sex, self-rated health, participation in activities provided by organizations or societies, educational level, household income per consumption unit, low/high MHI-5 score, and an index of social relationships (see previous section for detailed description). The estimates of the covariates are provided as Supplemental Material (Appendix Table A.1). To assess the first study objective, the predicted proportions were calculated separately for people living alone and for those not living alone. In response to the second objective, the predicted proportions were calculated for the interaction between living alone (vs not) and sex, age group, MHI-5, BDI-6, and 3 levels of WEMWBS. When the results were stratified according to BDI-6 or WEMWBS, the adjustment model did not include MHI-5 due to their strong correlation.

To address any potential response bias and to ensure representativeness, the models also took the stratified sampling design into account and non-participation was adjusted using sampling weights. The weights were calculated using socio-demographic variables (gender, age, official language, marital status, geographic area, and most recent employment and unemployment) and information about hospitalizations/treatments for a number of health conditions (cardiovascular diseases, mental health diagnoses, infections, birth and pregnancy, and accidents/poisonings/external causes), obtained from national administrative registers.^16^ Missing values were excluded. The differences in the predicted proportions were tested with 2-tailed tests, with “statistical significance” determined at *P* < .05.

As sensitivity analyses to confirm one of our key results, we used the same methodology to explain having seen a doctor more than once and more than twice in the past 12 months.

## Results

### Descriptive information

Reflecting the Finnish population,^2^ those living alone were more often females and aged 65 or more, compared with those not living alone (Table 1). Among those living alone, compared with those not living alone, it was more common to experience poor/average health (45% vs 32%), have a high MHI5 (9% vs 6%) or BDI6 (9% vs 15%) score, or a low level of WEMWBS (20% vs 12%).

### Differences in service use and service evaluations between those living alone and not living alone

**Figure 1.**
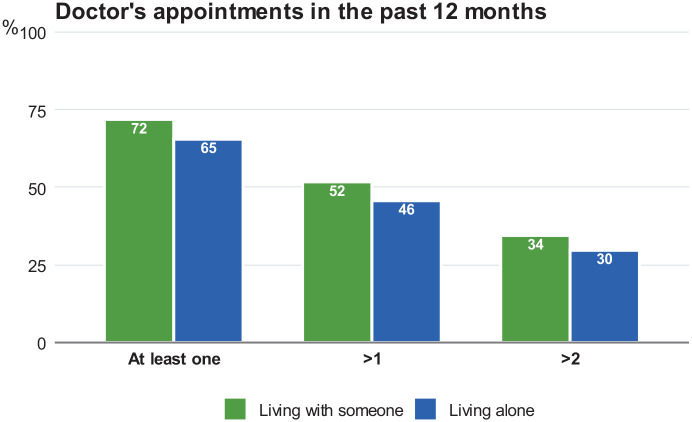
The predicted proportions of having seen a doctor at least once, twice, and three times in the past 12 months among people living alone and with someone (adjusted for age, sex, self-rated health, participation in organizations or societies, education, household income, and social relationship index).

Regarding the use of health services, those who lived alone had been to a doctor’s appointment at least once in the past 12 months less often (65.5%) than those not living alone (71.9%; Table 2). As an examination on the sensitivity of these results, we found similar patterns with having seen a doctor more than once and more than twice (Figure 1). Having seen a nurse and having used health services for mental health issues were equally common between those living alone and with someone. On the contrary, people living alone had less often had a health examination in the past 5 years (72.4%) than those not living alone (79.2%). This difference was greater in the youngest age group, 18- to 29-year-olds (67.5% and 86.8%, respectively), than in the older ones (74.2% vs 75.8% among the 30-64-year-olds, and 75.2% and 83.5% among those aged 65 or more).

**Table 2.** Adjusted proportions and 95% confidence intervals (CI) of use and experiences of health services among people living with someone and alone, main effects and stratification by sex and age group.

	Not living alone, % (CI)	Living alone, % (CI)	*P*-value (main effect)	*P*-value (interaction)^a^
Doctor’s appointment in the past 12 mo				
All	71.9 (70.0, 73.8)	65.5 (61.6, 69.4)	.002	
Sex				
Men	66.4 (63.3, 69.4)	59.7 (54.2, 65.3)	<.001	.866
Women	77.0 (74.5, 79.6)	70.8 (64.9, 76.7)		
Age				
18-29	65.4 (56.7, 74.1)	54.6 (41.5, 67.7)	.077	.365
30-64	72.3 (70.3, 74.4)	69.8 (65.9, 73.7)		
65-99	75.8 (72.4, 79.2)	69.2 (64.0, 74.4)		
Nurse’s appointment in the past 12 mo				
All	48.4 (46.4, 50.5)	47.7 (44.0, 51.4)	.727	
Sex				
Men	45.0 (42.2, 47.9)	46.2 (40.2, 52.3)	.004	.450
Women	51.5 (48.4, 54.7)	49.2 (43.7, 54.7)		
Age				
18-29	50.4 (41.5, 59.4)	45.1 (32.4, 57.8)	.866	.749
30-64	48.1 (45.8, 50.3)	48.8 (44.8, 52.7)		
65-99	47.8 (44.1, 51.4)	48.3 (42.8, 53.8)		
Used health services because of mental health problems in the past 12 mo				
All	8.7 (7.2, 10.3)	10.6 (8.0, 13.3)	.181	.181
Sex				
Men	6.2 (4.2, 8.1)	8.5 (5.5, 11.6)	.001	.567
Women	10.9 (8.8, 13.0)	12.4 (8.2, 16.5)		
Age				
18-29	15.6 (8.9, 22.2)	15.7 (6.9, 24.5)	<.001	.526
30-64	9.0 (7.6, 10.4)	12.7 (10.1, 15.3)		
65-99	2.8 (1.5, 4.2)	3.0 (1.4, 4.6)		
Health examination in the past 5 y				
All	79.2 (77.5, 81.0)	72.4 (68.9, 76.0)	.001	
Sex				
Men	82.5 (80.2, 84.8)	76.0 (71.4, 80.6)	<.001	.852
Women	76.1 (73.8, 78.4)	69.0 (63.4, 74.6)		
Age				
18-29	86.8 (81.2, 92.5)	67.5 (55.0, 80.0)	<.001	.013
30-64	75.8 (73.6, 78.1)	74.2 (70.2, 78.2)		
65-99	83.5 (80.8, 86.2)	75.2 (70.9, 79.6)		
Positive experiences of primary care				
All	55.8 (53.3, 58.3)	51.7 (47.1, 56.2)	.108	
Sex				
Men	58.5 (54.7, 62.4)	54.3 (48.4, 60.3)	.032	.964
Women	53.5 (50.6, 56.4)	49.4 (42.9, 56.0)		
Age				
18-29	61.3 (51.0, 71.7)	39.2 (24.7, 53.7)	<.001	.058
30-64	52.0 (49.6, 54.5)	52.7 (48.1, 57.3)		
65-99	61.9 (58.6, 65.2)	61.4 (55.6, 67.2)		
Positive experiences of the access to primary care				
All	56.1 (53.2, 59.0)	48.7 (43.5, 53.9)	.008	
Sex				
Men	59.8 (56.0, 63.7)	51.5 (43.4, 59.5)	.009	.742
Women	53.0 (49.2, 56.8)	46.4 (39.8, 53.1)		
Age				
18-29	53.5 (41.3, 65.7)	37.9 (21.3, 54.5)	.013	.394
30-64	54.0 (50.8, 57.1)	50.6 (44.8, 56.5)		
65-99	62.0 (57.3, 66.7)	54.8 (48.5, 61.1)		

All predicted proportions adjusted for age, sex, self-rated health, participation in organizations or societies, education, household income, low/high MHI-5 score, and social relationship index.

a Interactions with living alone.

Considering assessments of primary care and its accessibility, those who lived with someone had better experiences of the access to primary care (56.1%) than those living alone (48.7%), but there were no differences in how primary care services were evaluated (evaluations were positive for 55.8% among those living with someone and 51.7% among those living alone; Table 2).

### Subgroup analyses by gender and age

The data did not give evidence that the differences in men and women would vary according to household composition in terms of any of the outcomes (Table 2). There was a weak indication that young people living alone had less positive experiences of primary care (positive experience reported by 39.2%) than young people living with someone (61.3%), whereas in other age groups these evaluations did not differ (52.0%-52.7% among 30-64-year-olds and 61.4%-61.9% among those aged 65 or more).

### Subgroup analyses by different mental health indicators

The adjusted proportions based on MHI5, BDI6, and WEMWBS in Table 3 show that for people living alone, having had a health examination in the past 5 years was more unusual for those scoring high on the BDI6 scale (59.0%) compared to lower scores (75.0%). On the other hand, among people living with someone the respective difference was small (77.0% for high and 79.8% for low MHI5 level). This trend was similar across different levels of MHI5 and WEMWBS, albeit the test statistics were not “significant.” No other differences between people living alone versus not living alone were found in terms of BDI and the other outcomes, or between different levels of MHI5 or WEMWBS and any of the outcomes.

**Table 3.** Adjusted proportions and 95% confidence intervals (CI) of use and experiences of health services among people living with someone and alone, stratified by mental health indicators.

	Not living alone, % (CI)	Living alone, % (CI)	*P*-value^a^
Doctor’s appointment in the past 12 mo			
Psychological distress (MHI5)			
Low	71.5 (69.5, 73.4)	64.8 (60.6, 69.0)	.780
High	81.4 (75.0, 87.9)	78.3 (67.0, 89.6)	
Depressive symptoms (BDI6)			
Low	71.1 (69.0, 73.2)	64.8 (60.4, 69.1)	.851
High	79.3 (73.8, 84.9)	75.1 (67.0, 83.1)	
Positive mental health (WEMWBS)			
Low	75.6 (70.5, 80.6)	76.8 (70.7, 82.9)	.232
Average	71.9 (69.7, 74.1)	63.8 (58.8, 68.9)	
High	68.0 (62.2, 73.7)	62.5 (52.4, 72.5)	
Nurse’s appointment in the past 12 mo			
Psychological distress (MHI5)			
Low	48.0 (45.8, 50.1)	46.7 (42.8, 50.5)	.406
High	56.1 (47.2, 65.0)	61.3 (48.4, 74.2)	
Depressive symptoms (BDI6)			
Low	47.4 (45.2, 49.7)	45.8 (41.8, 49.7)	.190
High	55.7 (48.5, 62.9)	61.0 (52.8, 69.2)	
Positive mental health (WEMWBS)			
Low	52.4 (46.3, 58.6)	56.2 (48.6, 63.8)	.567
Average	48.3 (45.7, 50.9)	46.1 (41.4, 50.8)	
High	43.4 (38.1, 48.7)	45.1 (35.9, 54.4)	
Used health services because of mental health problems in the past 12 mo			
Psychological distress (MHI5)			
Low	6.6 (5.1, 8.1)	8.2 (5.3, 11.0)	.815
High	29.6 (22.6, 36.7)	36.0 (25.0, 47.1)	
Depressive symptoms (BDI6)			
Low	6.0 (4.6, 7.5)	7.2 (4.4, 10.0)	.359
High	23.5 (17.7, 29.2)	33.0 (25.6, 40.4)	
Positive mental health (WEMWBS)			
Low	18.9 (14.4, 23.4)	25.5 (19.4, 31.6)	.188
Average	6.7 (5.2, 8.2)	7.5 (4.4, 10.7)	
High	5.0 (2.6, 7.4)	1.8 (0^b^, 5.1)	
Health examination in the past 5 y			
Psychological distress (MHI5)			
Low	79.4 (77.5, 81.3)	73.6 (69.8, 77.4)	.175
High	77.2 (71.7, 82.6)	60.7 (48.5, 72.9)	
Depressive symptoms (BDI6)			
Low	79.8 (77.9, 81.7)	75.0 (71.3, 78.7)	**.025**
High	77.0 (72.1, 82.0)	59.0 (50.1, 67.9)	
Positive mental health (WEMWBS)			
Low	77.6 (73.3, 81.9)	62.8 (55.8, 69.8)	.056
Average	79.6 (77.6, 81.6)	74.1 (69.6, 78.5)	
High	80.0 (75.4, 84.6)	79.6 (72.6, 86.5)	
Positive experiences of primary care			
Psychological distress (MHI5)			
Low	56.2 (53.6, 58.8)	50.8 (46.3, 55.4)	.187
High	49.7 (40.9, 58.6)	56.7 (41.1, 72.2)	
Depressive symptoms (BDI6)			
Low	56.1 (53.3, 58.8)	52.6 (47.8, 57.4)	.614
High	52.4 (44.3, 60.5)	44.4 (31.3, 57.5)	
Positive mental health (WEMWBS)			
Low	42.4 (36.6, 48.2)	45.6 (35.2, 55.9)	.189
Average	55.8 (52.8, 58.7)	50.1 (44.8, 55.5)	
High	68.3 (62.5, 74.1)	72.2 (61.9, 82.6)	
Positive experiences of the access to primary care			
Psychological distress (MHI5)			
Low	56.9 (53.8, 60.0)	48.3 (42.8, 53.9)	.204
High	45.6 (35.3, 55.9)	49.5 (33.6, 65.3)	
Depressive symptoms (BDI6)			
Low	58.3 (55.4, 61.1)	50.5 (45.0, 56.1)	.307
High	43.1 (32.6, 53.6)	43.3 (32.8, 53.7)	
Positive mental health (WEMWBS)			
Low	39.5 (32.8, 46.2)	39.3 (30.8, 47.9)	.506
Average	57.1 (53.5, 60.6)	49.7 (43.2, 56.3)	
High	70.9 (65.0, 76.9)	71.9 (59.6, 84.2)	

All predicted proportions adjusted for age, sex, self-rated health, participation in organizations or societies, education, household income, and social relationship index.

a *P*-values are for the interactions with living alone.

b Lower bound of the CI, based on normality assumption, was negative (−1.6).

## Discussion

Our first study objective was to assess the use and evaluation of health services in people living alone and with someone. We found that although people living alone used some of the assessed health services equally often than those living with someone, the use of the most common services, that is, seeing a doctor and having a basic health examination, were less common. These differences held even when controlling for their differences in general and mental health (ie, the potential need for health services). The second objective was to examine whether some subgroups within people living alone use health services less than others or evaluate health services more negatively. We found that among those living alone with a high level of depressive symptoms, having had a health examination was rarer than among those who lived with someone and had a high level of depressive symptoms. Those with depressive symptoms are, thus, a potential subgroup of people living alone that receive less preventive care, although their other service use rates were similar to those living with someone. Similarly, in the youngest age group, those living alone had been to a health examination less often than those living with someone. Otherwise our results indicated that within people living alone, neither female or male gender nor the other age groups would be in the risk of receiving less health support than the respective group among those living with other people.

Consistent with Terämä et al,^7^ our study found that those living alone use most public health services almost as much as those living with a partner. The difference between the uses of doctor’s services in our study might be attributed to lower usage of private health care (including occupational health services) among those living alone, as was suggested in the report by Terämä et al^7^ The report proposed that among those living alone, there were more people with low income and/or unemployment, and therefore they may have poorer possibilities to complement public health services with occupational or private health care. As our study did not differentiate between public and private sectors, we could not explore this hypothesis further. Furthermore, earlier studies^1,2,25^ suggest that spousal support increased the use of medical services. Thus, it could be suggested, that those living with someone, especially solo-dwelling elderly, benefit from having another person in the household.

The number of people living alone is increasing globally.^26^ As the world is also currently aging,^27^ it creates a new pressure to social and health care services, as living in one’s own home as long as possible is needed. Health services should be flexible enough to provide the required services at-home.^27^ At the same time, they should acknowledge the vulnerability^3-6^ of those living alone.

Limitations of this study include the usage of cross-sectional data which prohibits making any causal conclusions. Furthermore, all measures were self-reported and thus subjective to same-source bias. Linking the responses with the registry-obtained information from health care usage would have potentially reduced some of this bias, and we recommend prospective studies to consider this option.

Despite of the limitations, this study has increased our knowledge of the use of health services of this population group emphasizing that those living alone must be acknowledged as a vulnerable group needing focused attention.

## Conclusions

Based on a nationally representative health survey, our results indicate that those living alone use some health services less frequently compared to those living with someone. Concerning regular check-ups, the difference is greater in the younger age groups than in the older. Further research is needed to find out the reason for this. Our results suggest that concerning the use of health services, it is important to acknowledge all age groups among those living alone, and not just assume that the potentially adverse issues related to living alone apply solely to the elderly. Especially those living alone who are suffering from symptoms of depression might be a potential group that does not receive the same levels of preventive care than others and thus should be given special attention.

Those living with a partner seemed to have more positive experiences of the access to primary care. Our results did not reveal a reason for this; however, it would be an interesting topic of further studies.

## Supplemental Material

sj-docx-1-his-10.1177_11786329211043955 – Supplemental material for Use of Health Services Among People Living Alone in Finland Click here for additional data file. Supplemental material, sj-docx-1-his-10.1177_11786329211043955 for Use of Health Services Among People Living Alone in Finland by Pia CM Solin, Tytti P Pasanen, Katariina AJ Mankinen, Tuija P Martelin and Nina M Tamminen in Health Services Insights
