# Supplementary material for: Use of Health Services Among People Living Alone in Finland
Source: Health Serv Insights. 2021 Sep 1;14:11786329211043955. doi: 10.1177/11786329211043955 (PMC8414606; doi:10.1177/11786329211043955)
Supplement: sj-docx-1-his-10.1177_11786329211043955 – Supplemental material for Use of Health Services Among People Living Alone in Finland [file sj-docx-1-his-10.1177_11786329211043955.docx]

Appendix Table A.1 Odds ratio estimates and 95% CIs of the logistic regression model used to calculate the reported predicted proportions

|  | Doctor's appointment in the past 12 months (n=4686) | | Nurse's appointment in the past 12 months (n=4691) | | Health examination in the past 5 years (n=4673) | | Used health services because of mental health problems in the past 12 months (n=4680) | | Positive experiences of primary care (n=3641) | | Positive experiences of the access to primary care (n=2514) | |
| --- | --- | --- | --- | --- | --- | --- | --- | --- | --- | --- | --- | --- |
|  | Exp(b)  [CI] | p | Exp(b)  [CI] | p | Exp(b)  [CI] | p | Exp(b)  [CI] | p | Exp(b)  [CI] | p | Exp(b)  [CI] | p |
| Age | 1.01  [1.01; 1.02] | <.001 | 1  [1; 1.01] | .635 | 1.01  [1; 1.01] | .021 | .96  [.95; .97] | <.001 | 1.01  [1; 1.02] | .002 | 1.01  [1.01; 1.02] | .002 |
| Sex (female vs male) | 1.74  [1.4; 2.16] | <.001 | 1.26  [1.05; 1.51] | .011 | .68  [.57; .8] | <.001 | 1.89  [1.35; 2.63] | <.001 | .81  [.68; .96] | .014 | .76  [.62; .93] | .008 |
| Self-rated health: Rather good vs good | 1.58  [1.23; 2.03] | <.001 | 1.55  [1.26; 1.92] | <.001 | 1.15  [.93; 1.41] | .187 | 1.6  [.99; 2.59] | .057 | .69  [.55; .87] | .002 | .6  [.44; .82] | .001 |
| Self-rated health: Average vs good | 2.5  [1.87; 3.34] | <.001 | 2.05  [1.61; 2.62] | <.001 | .93  [.71; 1.2] | .559 | 1.95  [1.21; 3.13] | .006 | .53  [.41; .68] | <.001 | .39  [.27; .56] | <.001 |
| Self-rated health: Rather poor vs good | 6.15  [3.58; 1.57] | <.001 | 2.78  [2.01; 3.85] | <.001 | .77  [.5; 1.19] | .24 | 3.02  [1.65; 5.51] | <.001 | .29  [.21; .41] | <.001 | .23  [.15; .36] | <.001 |
| Self-rated health: Poor vs good | 9.41  [2.57; 34.51] | <.001 | 4.07  [1.67; 9.93] | .002 | .54  [.27; 1.1] | .089 | 3.03  [1.29; 7.1] | .011 | .69  [.36; 1.34] | .277 | .36  [.17; .77] | .009 |
| Participation in societies (sometimes vs never) | 1.2  [.91; 1.57] | .194 | 1.22  [.99; 1.5] | .068 | 1.04  [.85; 1.27] | .715 | 1.07  [.67; 1.73] | .765 | .98  [.77; 1.25] | .887 | .78  [.59; 1.04] | .089 |
| Participation in societies (often vs never) | 1.29  [1.05; 1.58] | .016 | 1.22  [1.01; 1.47] | .037 | 1.11  [.92; 1.35] | .279 | .66  [.45; .96] | .031 | 1.07  [.88; 1.3] | .503 | 1.01  [.78; 1.3] | .95 |
| Education (medium vs low) | 1.04  [.81; 1.34] | .759 | 1.06  [.87; 1.29] | .552 | .86  [.7; 1.05] | .13 | 1.22  [.77; 1.92] | .396 | .94  [.73; 1.19] | .589 | 1.21  [.93; 1.57] | .16 |
| Education (high vs low) | 1  [.77; 1.3] | .996 | .93  [.76; 1.13] | .468 | .73  [.59; .9] | .003 | 1.91  [1.24; 2.94] | .003 | 1.18  [.94; 1.47] | .15 | 1.27  [.94; 1.7] | .12 |
| Household income (in thousands, per consumption unit) | 1.01  [1.01; 1.02] | <.001 | 1  [.99; 1] | .583 | 1.01  [1; 1.01] | .002 | .99  [.97; 1] | .012 | 1.01  [1; 1.01] | .003 | 1.01  [1.01; 1.02] | <.001 |
| MHI5 (low vs high) | 1.84  [1.21; 2.78] | .004 | 1.54  [1.09; 2.19] | .014 | .7  [.51; .96] | .027 | 7.4  [5.05; 1.84] | <.001 | .91  [.62; 1.31] | .6 | .73  [.47; 1.12] | .152 |
| Social relationship index | .96  [.92; 1] | .059 | .95  [.93; .98] | <.001 | .95  [.92; .97] | <.001 | .99  [.93; 1.04] | .625 | .96  [.93; 1] | .055 | .98  [.95; 1.02] | .4 |
